# Long non-coding RNAs towards precision medicine in gastric cancer: early diagnosis, treatment, and drug resistance

**DOI:** 10.1186/s12943-020-01219-0

**Published:** 2020-05-27

**Authors:** Li Yuan, Zhi-Yuan Xu, Shan-Ming Ruan, Shaowei Mo, Jiang-Jiang Qin, Xiang-Dong Cheng

**Affiliations:** 1 The First Affiliated Hospital of Zhejiang Chinese Medical University, Hangzhou, 310006 China; 2 Institute of Cancer and Basic Medicine, Chinese Academy of Sciences, Cancer Hospital of the University of Chinese Academy of Sciences, Zhejiang Cancer Hospital, Banshan Road 1#, Gongshu District, Hangzhou, 310022 China; 3 College of Pharmaceutical Sciences, Zhejiang Chinese Medical University, 548 Binwen Road, Binjiang District, Hangzhou, 310053 China

**Keywords:** LncRNA, Gastric cancer, Precision medicine, Early diagnosis, Cancer treatment, Chemoresistance

## Abstract

Gastric cancer is a deadly disease and remains the third leading cause of cancer-related death worldwide. The 5-year overall survival rate of patients with early-stage localized gastric cancer is more than 60%, whereas that of patients with distant metastasis is less than 5%. Surgical resection is the best option for early-stage gastric cancer, while chemotherapy is mainly used in the middle and advanced stages of this disease, despite the frequently reported treatment failure due to chemotherapy resistance. Therefore, there is an unmet medical need for identifying new biomarkers for the early diagnosis and proper management of patients, to achieve the best response to treatment. Long non-coding RNAs (lncRNAs) in body fluids have attracted widespread attention as biomarkers for early screening, diagnosis, treatment, prognosis, and responses to drugs due to the high specificity and sensitivity. In the present review, we focus on the clinical potential of lncRNAs as biomarkers in liquid biopsies in the diagnosis and prognosis of gastric cancer. We also comprehensively discuss the roles of lncRNAs and their molecular mechanisms in gastric cancer chemoresistance as well as their potential as therapeutic targets for gastric cancer precision medicine.

## Background

Gastric cancer is one of the most common malignancies worldwide, with more than one million new cases every year, and remains the third leading cause of cancer-related deaths [1, 2]. The clinical stage at the time of diagnosis directly determines the prognosis of patients with this disease. The patients with localized, early-stage gastric cancer usually have a high 5-year overall survival (OS) rate (> 60%), whereas the 5-year OS rates for gastric cancer patients with local and distant metastasis dramatically decrease to 30 and 5%, respectively [2]. Unfortunately, due to the occult and atypical nature of early clinical symptoms of gastric cancer, more than 60% of patients have local or distant metastases at the time of diagnosis [2]. For patients with early gastric cancer, surgical resection is the best treatment option; for patients who cannot undergo surgical resection or patients with advanced metastases, chemotherapy is the most important treatment [3, 4]. However, poor or even no response to chemotherapy is often observed in gastric cancer patients because of the intrinsic or acquired resistance, which becomes the most common cause of treatment failure [5]. Therefore, the low rate of early diagnosis and chemotherapy resistance constitute the main contributions to the poor prognosis of gastric cancer.

To date, the biomarkers commonly used in early screening for gastric cancer include carcinoembryonic antigen (CEA), alpha-fetoprotein (AFP), carbohydrate antigen 19–9 (CA19–9), CA72–4, CA125, etc. [6, 7]. However, the sensitivities and positive rates of these biomarkers are poor; their sensitivities in the diagnosis of gastric cancer are from 4.7 to 33.3%, and the positive rates of CEA, CA199, and CA724 only range from 21.1 to 30% [7–9]. The diagnosis of gastric cancer still depends on upper gastrointestinal endoscopy, but its clinical application is limited because of the invasiveness and high cost [10]. Therefore, there is an urgent need for minimal-invasive or non-invasive detection approaches, as well as highly specific biomarkers, to improve gastric cancer early diagnosis and survival outcomes.

Long non-coding RNAs (lncRNAs) have attracted increasing attention as cancer biomarkers for early screening, diagnosis, prognosis, and responses to drug treatment [11–13]. A recent study has shown that the expression of lncRNA MNX1-AS1 (MNX1 antisense RNA 1) is significantly increased in gastric cancer tissues and associated with the poor prognosis of gastric cancer patients [14]. LncRNA SNHG11 (small nucleolar RNA host gene 11) has been reported as a potential biomarker for early detection of colon cancer and a new therapeutic target of this disease [15]. A stroma-related lncRNA panel has been found to predict recurrence and adjuvant chemotherapy benefit in patients with early-stage colon cancer [16]. LncRNAs are involved in the acquired resistance to chemotherapy [17, 18], and targeting lncRNA can reverse drug resistance and enhance the sensitivity of cancer cells to chemotherapy [19]. Given the importance of lncRNAs in cancer, a better understanding of their roles in the early diagnosis, treatment, prognosis, and drug resistance of gastric cancer may provide new insights for precise treatment and individualized management of patients with this disease.

The regulation of lncRNA expression and the roles of lncRNAs in gastric cancer progression and metastasis have been extensively discussed in several recent reviews [20–24]. In the present review, we focus on the clinical evidence of lncRNAs as biomarkers in liquid biopsies in the early diagnosis and prognosis of gastric cancer. We also comprehensively discuss the roles of lncRNAs and their molecular mechanisms in gastric cancer chemoresistance, as well as their potential as therapeutic targets for gastric cancer precise medicine.

### An overview of lncRNAs

The Encyclopedia of DNA Elements (ENCODE) project has revealed that only about 1.2% of human transcripts (RNAs) encode proteins and more than 98% of human transcripts are non-protein-coding RNAs (ncRNAs), such as lncRNAs, circular RNAs (circRNAs), microRNAs (miRNAs), and small nucleolar RNAs (snoRNAs) [25]. LncRNAs are the transcripts of more than 200 nucleotides, accounting for 80 to 90% of all ncRNAs and are characterized by low expression levels, poor interspecies conservation, and high expression coefficient of variance [26, 27].

According to their genomic localization and evolutionary lineage, lncRNAs can be divided into intergenic lncRNAs, intronic lncRNAs, exonic lncRNAs, sense lncRNAs, and antisense lncRNAs. Intergenic lncRNAs (also called lincRNAs) are transcribed from genomic regions between coding genes, while intronic lncRNAs overlap entirely with introns of protein-coding genes and exonic lncRNAs overlap entirely or partially with exons of protein-coding genes [28, 29]. The transcriptional orientation of lncRNAs can be in sense or antisense when compared with the transcriptional orientation of the protein-coding genes [30]. Besides, lncRNAs can be classified into nuclear lncRNAs and cytoplasmic lncRNAs based on the subcellular localization, which is critical for their functions. Most lncRNAs are located in the nucleus and only about 15% are in the cytoplasm [31]. Nuclear lncRNAs mainly regulate the transcription or mRNA processing, e.g. lncRNA XIST (X inactive specific transcript), MALAT1 (metastasis associated lung adenocarcinoma transcript 1), and NEAT1 (nuclear paraspeckle assembly transcript 1) functioning as transcription regulators [32–34]. Cytoplasmic lncRNAs are more often involved in post-transcriptional regulation, such as playing the role of miRNA sponges. Du et al. have demonstrated that cytoplasmic localization is an important factor in determining the sponge efficacy of lncRNA TUG1 (taurine up-regulated 1) [35]. Cytoplasmic lncRNA PVT1 (plasmacytoma variant translocation 1) has been found to act as a competitive endogenous RNA (ceRNA) against miR-214-3p and promote the progression of colon cancer [36].

LncRNAs were initially considered as “junk” or “genomic dark matter” without function. With the deepening of research in recent years, lncRNAs have been found to participate widely in various physiological and pathological processes of organisms. In the human body, lncRNAs not only regulate the physiological processes such as cell proliferation, differentiation, and apoptosis but also participate in regulating various pathological processes of the body, such as cancer, cardiovascular diseases, autoimmune diseases, diabetes, and more [37–40]. The specific function of lncRNAs is to regulate gene expression at the pre-transcriptional, transcriptional, and post-transcriptional levels. At the pre-transcriptional level, lncRNA regulates gene expression by gene modification, histone modification, and chromatin remodeling, without changing the DNA sequences of the organisms [41, 42]. During transcription, lncRNA interacts with transcription factors to regulate gene transcription [43]. At the post-transcriptional level, lncRNA acts as a precursor of some miRNAs to regulate gene expression, or as a ceRNA to regulate the translation of the corresponding mRNA [44]. However, due to the large number of lncRNAs, the functions of most lncRNAs are still unclear and require further comprehensive research.

### LncRNAs as liquid biopsy biomarkers of gastric cancer

The development of liquid biopsies has opened a new era for precision medical treatment of human cancer. Because of their minimal-invasive or non-invasive characteristics and high public acceptance, liquid biopsies can be conducted more frequently for early screening, diagnosis, and prognosis of cancer. Besides, liquid biopsies can be collected at specific time intervals to monitor responses to treatment, drug resistance, recurrence, and metastasis of cancer. Added benefits are that, unlike tissue biopsies obtained from only one tumor area, liquid biopsies may better reflect the genetic characteristics of all tumor subclones in patients [45]. LncRNAs are widely distributed in peripheral plasma/serum, saliva, gastric juice, urine, semen, and other liquids and play important roles in various aspects of human physiological and pathological processes [46–50]. Based on the aforementioned benefits, a comprehensive understanding of the current research status of lncRNAs is critical for the further development of them as cancer biomarkers in liquid biopsies.

Accumulating evidence suggests the usefulness of lncRNAs as liquid biopsy biomarkers for human cancer. LncRNAs in peripheral blood plasma/serum have been demonstrated as biomarkers for various types of human cancer, such as lung, breast, and colon cancer [51–53]. LncRNAs in saliva have been mainly used as biomarkers for head and neck cancer, such as oral, pharyngeal, and laryngeal cancer [54, 55]. LncRNAs in gastric juice and urine have also been reported as biomarkers of gastric cancer and urinary system cancer, respectively [49]. Of note, the urinary level of lncRNA PCA3 (prostate cancer associated 3) has been used as a biomarker for the diagnosis of prostate cancer in clinical applications [56, 57]. Although there is no report on lncRNAs in semen, recent studies have shown that miRNAs in semen may be used as biomarkers for prostate cancer [58]. To date, almost all attention has been paid to the lncRNAs in plasma/serum and gastric juice but not in other liquid biopsies as biomarkers of gastric cancer, which has been comprehensively discussed in this section.

### LncRNAs in plasma/serum as diagnostic and prognostic biomarkers of gastric cancer

The development of a disease often leads to changes in the plasma/serum composition, which can be detected to reflect the status of the disease [59]. LncRNAs, which are freely circulating in the plasma/serum or packaged in exosomes, have all of the characteristics of ideal biomarkers because they are stable over long periods at room temperature, during repeated freeze-thaw cycles, or at different pH values [60]. More importantly, the plasma/serum levels of lncRNAs are mostly the same as those in the primary tumor tissues, thus precisely reflecting the characteristics of the tumors [61, 62]. In addition, the collection of plasma/serum samples at different time points is relatively convenient for monitoring the progress of the disease [63–66].

#### LncRNAs in plasma/serum as diagnostic biomarkers of gastric cancer

A large number of circulating lncRNAs have been reported as biomarkers for the diagnosis of gastric cancer (as summarized in Table 1), which have obvious advantages over the diagnostic biomarkers in clinical applications. Xian et al. have found that lncRNA HULC (hepatocellular carcinoma upregulated long noncoding RNA) and ZNFX1-AS1 (ZNFX1 antisense RNA 1) can distinguish gastric cancer patients from healthy controls and have proposed them as biomarkers for diagnosing gastric cancer [77]. The receiver operator characteristic curve (ROC) analysis has shown that the area under curve (AUC) values for HULC and ZNFX1-AS1 are 0.65 and 0.85, respectively, which are higher than those of traditional serum biomarkers, including CEA (0.62), CA19–9 (0.56), CY211 (0.59), and neuron-specific enolase (NSE, 0.56) [77]. Jin et al. have further confirmed that HULC is more sensitive and specific than CEA and CA724 as a diagnostic marker of gastric cancer [82]. Yang et al. have found that the AUC values of lncRNA PANDAR (promoter of CDKN1A antisense DNA damage activated RNA), FOXD2-AS1 (FOXD2 adjacent opposite strand RNA 1), and SMARCC2 (SWI/SNF related, matrix associated, actin dependent regulator of chromatin subfamily c member 2) as diagnostic biomarkers of gastric cancer are 0.77, 0.7, and 0.75, respectively, which are similar to the AUC value of combined CEA, AFP, CA125, CA153, and CA199 [97]. Feng et al. have also demonstrated that lncRNA B3GALT5-AS1 (B3GALT5 antisense RNA 1) is better than CEA and CA19–9 as a diagnostic biomarker of gastric cancer [87]. Zhou et al. have recently reported that lncRNA C5orf66-AS1 (C5orf66 antisense RNA 1) can be utilized for the diagnosis of gastric cancer with the AUC value of 0.688 [67]. More importantly, lncRNA C5orf66-AS1 has further been shown to predict early gastric cancer with the AUC value of 0.789 [67].

**Table 1:** LncRNAs in plasma/serum as diagnostic and prognostic biomarkers of gastric cancer

LncRNA	Biomarker type	Expression	Cases	Sensitivity	Specificity	AUC	Sample	Refs
C5orf66-AS1	Diagnostic	Down	200 patients with GC and 278 non-GC	77.5%	53.6%	0.688	Serum	[67]
PTCSC3	Diagnostic/Prognostic	Down	68 patients with GC and 60 healthy controls	N/A	N/A	0.92	Plasma	[68]
ARHGAP27P1	Diagnostic/Prognostic	Down	53 patients with GC and 53 healthy controls	75.5%	60.4%	0.732	Plasma	[61]
TUBA4B	Diagnostic/Prognostic	Down	37 patients with GC and 37 healthy controls	N/A	N/A	0.8075	Plasma	[69]
LINC00086	Diagnostic/Prognostic	Down	168 patients with GC and 74 healthy controls	72.6%	83.8%	0.860	Plasma	[70]
DGCR5	Diagnostic/Prognostic	Down	34 patients with GC and 34 healthy controls	N/A	N/A	0.722	Plasma	[71]
SNHG17	Diagnostic/Prognostic	Up	67 patients with GC and 67 healthy controls	N/A	N/A	0.748	Plasma	[72]
MEF2C-AS1	Diagnostic	Down	46 patients with GC and 21 healthy controls	N/A	N/A	0.733	Plasma	[73]
MT1JP	Diagnostic/Prognostic	Down	34 patients with GC and 34 healthy controls	N/A	N/A	0.649	Plasma	[64]
GACAT2	Diagnostic/Prognostic	Up	117 patients with GC and 80 healthy controls	87.2%	28.2%	0.622	Plasma	[74]
RMRP	Diagnostic	Down	83 patients with GC and 90 healthy controls	59.1%	67.8%	0.693	Plasma	[49]
UCA1	Diagnostic	Up	20 patients with GC and 20 healthy controls	89.2%	80.3%	0.928	Plasma	[75]
LINC00152	Diagnostic	Up	79 patients with GC and 81 healthy controls	48.1%	85.2%	0.657	Plasma	[76]
ZNFX1-AS1	Diagnostic	Up	50 patients with GC and 50 healthy controls	84%	68%	0.85	Plasma	[77]
GASL1	Diagnostic	Down	112 patients with GC and 56 healthy controls	N/A	N/A	N/A	Plasma	[78]
GASL1	Diagnostic/Prognostic	Down	88 patients with GC and 72 healthy controls	N/A	N/A	0.8945	Serum	[79]
MALAT1	Diagnostic/Prognostic	Up	64 patients with GC and 64 healthy controls	N/A	N/A	0.8984	Plasma	[80]
MALAT1	Prognostic	Up	36 GC/NDM and 36 GC/DM	N/A	N/A	N/A	Plasma	[81]
HULC	Diagnostic	Up	50 patients with GC and 50 healthy controls	58%	80%	0.65	Plasma	[77]
HULC	Diagnostic	Up	100 patients with GC and110healthy controls	82%	83.6%	0.888	Serum	[82]
H19	Diagnostic	Up	43 patients with GC and 34 healthy controls	74%	58%	0.64	Plasma	[83]
H19	Diagnostic	Up	70 patients with GC and 70 healthy controls	82.9%	72.9%	0.838	Plasma	[84]
H19	Diagnostic	Up	35 patients with GC and 25 healthy controls	90.9%	100%	0.982	Plasma	[85]
H19	Diagnostic	Up	40 patients with GC and 42 healthy controls	87.2%	37.2%	0.643	Plasma	[86]
B3GALT5-AS1	Diagnostic	Up	107 patients with GC and 87 healthy controls	87.4%	74.7%	0.816	Serum	[87]
HOXA11-AS	Diagnostic/Prognostic	Up	94 patients with GC and 40 healthy controls	78.7%	97.8%	0.924	Serum	[88]
SNHG6	Diagnostic/Prognostic	Up	114 patients with GC and 99 healthy controls	N/A	N/A	N/A	Serum	[89]
DANCR	Diagnostic/Prognostic	Up	55 patients with GC and 39 healthy controls	72.7%	79.5%	0.816	Serum	[90]
LINC00978	Diagnostic	Up	38 patients with GC and 31 healthy controls	80%	70%	0.831	Serum	[91]
ZFAS1	Diagnostic/Prognostic	Up	77 patients with GC and 60 healthy controls	76.6%	63.9%	0.727	Plasma	[92]
Exosomal ZFAS1	Diagnostic/Prognostic	Up	60 patients with GC and 37 healthy controls	71.7%	75.7%	0.792	Serum	[93]
Exosomal lncUEGC1	Diagnostic	Up	51 patients with GC and 60 healthy controls	N/A	N/A	0.8760	Plasma	[65]
Exosomal lncUEGC2	Diagnostic	Up	51 patients with GC and 60 healthy controls	N/A	N/A	0.7582	Plasma	[65]
Exosomal PCSK2–2:1	Diagnostic/Prognostic	Down	63 patients with GC and 29 healthy controls	84%	86.5%	0.896	Serum	[94]
Exosomal GNAQ-6:1	Diagnostic	Down	43 patients with GC and 27 healthy controls	83.7%	55.6%	0.732	Serum	[95]
Exosomal MIAT	Diagnostic/Prognostic	Up	109 patients with GC and 50 healthy controls	N/A	N/A	0.892	Serum	[96]
PANDAR	Diagnostic	Up	109 patients with GC and 106 healthy controls	N/A	N/A	0.767	Plasma	[97]
FOXD2-AS1		Up		N/A	N/A	0.700		
SMARCC2		Up		N/A	N/A	0.748		
Combined		N/A		N/A	N/A	0.839		
H19	Diagnostic	Up	62 patients with GC and 40 healthy controls	74.19%	90.0%	0.854	Plasma	[98]
MEG3		Down		95.16%	42.50%	0.638		
miR-675-5p		Up		77.42%	52.50%	0.661		
Combined		N/A		88.87%	85%	0.927		
CTC-501O10.1	Diagnostic	Up	100 patients with GC and 100 healthy controls	90%	51%	0.74	Plasma	[99]
AC100830.4		Up		84%	58%	0.73		
RP11-210 K20.5		Up		89%	55%	0.737		
Combined		N/A		99%	49%	0.764		
INHBA-AS1	Diagnostic	Up	51 patients with GC and 53 healthy controls	N/A	N/A	0.855	Plasma	[100]
MIR4435–2HG		Up		N/A	N/A	0.882		
CEBPA-AS1		Up		N/A	N/A	0.785		
AK001058		Up		N/A	N/A	0.852		
Combined		N/A		N/A	N/A	0.921		
TINCR	Diagnostic	Up	80 patients with GC and 80 healthy controls	69%	56%	0.66	Plasma	[66]
CCAT2		Up		85%	51%	0.63		
AOC4P		Up		86%	41%	0.67		
BANCR		Up		75%	78%	0.81		
LINC00857		Up		93%	26%	0.61		
Combined		N/A		82%	87%	0.91		
FAM49B-AS	Diagnostic	Up	223 patients with GC and 223 healthy controls	N/A	N/A	0.609	Plasma	[101]
GUSBP11		Up		N/A	N/A	0.635		
CTDHUT		Up		N/A	N/A	0.762		
Combined		N/A		77.5%	73.9%	0.818		
United CA242, CA724		N/A		93.2%	86.6%	0.952		
H19	Diagnostic	Up	32 patients with GC and 30 healthy controls	68.75%	56.67%	0.724	Plasma	[102]
United CEA		N/A		N/A	N/A	0.804		
CTC-497E21.4	Diagnostic	Up	110 patients with GC and 84 healthy controls	81.82%	75.00%	0.848	Serum	[103]
United CEA, CA199		N/A		96.36%	42.86%	0.896		
Exosomal HOTTIP	Diagnostic	Up	126 patients with GC and 120 healthy controls	69.8%	85.0%	0.827	Serum	[104]
United CEA, CA199, CA724		N/A		N/A	N/A	0.870		

*N/A* Not available; *AUC* Area under curve; *GC* Gastric cancer

Circulating lncRNAs have better biomarker values when combined, e.g. combining lncRNA PANDAR, FOXD2-AS1, and SMARCC2 increases the AUC value to 0.84 [97]. The combination of lncRNA CTC-501O10.1, AC100830.4, and RP11-210 K20.5 has been found to improve the sensitivity of the diagnosis to 99% [99]. The combination of lncRNA INHBA-AS1 (INHBA antisense RNA 1), MIR4435–2HG (MIR4435–2 host gene), CEBPA-AS1 (CEBPA divergent transcript), and AK001058 has increased the AUC value to 0.92 [100]. Also, the combination of lncRNA TINCR (terminal differentiation-induced ncRNA), CCAT2 (colon cancer associated transcript 2), AOC4P (amine oxidase copper containing 4, pseudogene), BANCR (BRAF-activated non-protein coding RNA), and LINC00857 has increased the AUC value to 0.91, the sensitivity to 82%, and the specificity to 87% [66]. Meanwhile, combining lncRNAs and miRNAs have also improved their diagnostic efficiency, e.g. the AUC value of lncRNA H19 (H19 imprinted maternally expressed transcript) and MEG3 (maternally expressed 3) combined with miR-675-5p is 0.93 while the specificity and sensitivity are 88.9 and 85%, respectively [98]. However, the sensitivities of H19, MEG3, and miR-675-5p are 74.19, 95.16, and 77.42%, respectively, their respective specificities are 90.0, 42.50, and 52.50%, and their AUC values range from 0.638 to 0.854 [98]. Moreover, lncRNAs combined with traditional serum tumor markers have improved the diagnostic efficiency, e.g. lncRNA CTC-497E21.4 combined with CEA and CA199 has increased the AUC value to 0.9 [103]. Using the lncRNA FAM49B-AS (FAM49B antisense RNA), GUSBP11 (GUSB pseudogene 11), and CTDHUT (CTD highly upregulated transcript) combined with A242 and CA724, the AUC value, sensitivity, and specificity have been increased to 0.95, 93.2, and 86.6%, respectively [101].

#### LncRNAs in plasma/serum as prognostic biomarkers of gastric cancer

Tumor size, stage, depth of invasion, lymph node metastasis, distant metastasis, and pathological type are the relevant factors for the prognosis of cancer patients [105]. Circulating lncRNAs have been associated with these prognosis-related factors and have been demonstrated as prognostic biomarkers of gastric cancer (as summarized in Table 1). It has been found that the expression levels of lncRNA GASL1 (growth arrest associated lncRNA 1), PTCSC3 (papillary thyroid carcinoma susceptibility candidate 3), and MALAT1 are significantly correlated with tumor size, TNM (tumor, node, metastasis) stage, and distant metastasis of gastric cancer, respectively [68, 79, 80]. The expression levels of lncRNA SNHG6, ARHGAP27P1 (Rho GTPase activating protein 27 pseudogene 1), DANCR (differentiation antagonizing non-protein coding RNA), DGCR5 (DiGeorge syndrome critical region gene 5), MT1JP (metallothionein 1 J, pseudogene), SNHG17, and ZFAS1 (ZNFX1 antisense RNA 1) are closely related to the TNM stage, tumor invasion depth, and lymph node metastasis of gastric cancer [61, 64, 71, 72, 89, 90, 92]. It has also been reported that lncRNA HOXA11-AS (HOXA11 antisense RNA) and TUBA4B (tubulin alpha 4b) are tightly correlated with the tumor size, TNM stage, and lymph node metastasis of gastric cancer [69, 88]. Importantly, the Kaplan-Meier survival curve analysis has indicated that the patients with low expression of HOXA11-AS have a better survival rate, whereas the patients with low expression of TUBA4B have a shorter survival time [69, 88]. Tan et al. have demonstrated a significant correlation between the expression level of lncRNA GACAT2 (gastric cancer associated transcript 2) and the lymph node metastasis, distant metastasis, and perineural invasion of gastric cancer [74]. In addition, Ji et al. have shown that LINC00086 expression level is significantly associated with tumor size, lymph node metastasis, TNM stage, and the levels of CEA and CA19–9, while the gastric cancer patients with low expression of LINC00086 have low survival rates [70].

#### Exosomal lncRNAs in plasma/serum as diagnostic and prognostic biomarkers of gastric cancer

In the blood, long RNAs may be packaged into extracellular vesicles, which makes them more stable in plasma/serum. According to their diameters, the extracellular vesicles are classified into apoptotic bodies (50–5000 nm), microvesicles (50–1000 nm), and exosomes (30–100 nm) [106]. Apoptotic bodies are produced by cells undergoing programmed cell death, microvesicles are vesicles directly released from cell membranes, and exosomes are intracellular in origin [107, 108]. Among these types of vesicles, exosomes are the most abundant reservoir of lncRNAs [106]. Due to their intracellular origin and high quantities of long RNAs, circulating exosomal lncRNAs have been proposed as promising biomarkers for gastric cancer [109].

Compared with traditional diagnostic biomarkers (CEA, CA724, and CA199), circulating exosomal lncRNA PCSK2–2:1 (proprotein convertase subtilisin/kexin type 2–2:1) and GNAQ-6:1 (G protein subunit alpha q-6:1) have been reported as better biomarkers for distinguishing gastric cancer patients from healthy people. The AUCs (0.9 and 0.74, respectively), sensitivities (84 and 83.7%, respectively), and specificities (86.5 and 55.6%, respectively) of PCSK2–2:1 and GNAQ-6:1 are significantly better than the best traditional diagnostic biomarker CA724, which only has an AUC value of 0.57, a sensitivity of 56%, and a specificity of 65.5% [94, 95]. It has also been shown that the exosomal PCSK2–2:1 level is significantly related to the tumor size, TNM stage, and venous infiltration and may be developed as a prognostic biomarker of gastric cancer [94]. Lin et al. have found that the expression levels of exosomal lncRNA UEGC1 (ENST00000568893) and UEGC2 (ENST00000378432.1) are increased in patients with gastric cancer [65]. The stability tests have shown that almost all plasma UEGC1 is encapsulated by exosomes and has a higher AUC value while UEGC2 is only partially encapsulated by exosomes, suggesting that UEGC1 is more suitable to be developed as a diagnostic biomarker for early gastric cancer [65]. Xu et al. have shown that the serum level of exosomal lncRNA MIAT (myocardial infarction associated transcript) is significantly increased in gastric cancer patients, which is associated with worse clinical variables and shorter survival [96]. Moreover, it has been found that the serum exosomal MIAT is down-regulated in patients after treatment but markedly up-regulated in patients suffering recurrence [96]. Furthermore, exosomal lncRNAs combined with serum tumor markers have shown improved diagnostic accuracy, e.g.*,* exosomal lncRNA HOTTIP (HOXA distal transcript antisense RNA) combined with CEA, CA199, and CA724 have been found to increase the AUC value from 0.83 to 0.87 [104].

Taken together, lncRNAs in plasma/serum have shown great potential as biomarkers for the diagnosis and prognosis of gastric cancer. Importantly, the combinations, including but not limited to multiple lncRNAs combinations, lncRNA and miRNA combinations, and lncRNA and serum tumor marker combinations usually have better values as diagnostic biomarkers compared to an individual lncRNA. The exosomal lncRNAs in plasma/serum have also shown an advantage as biomarkers due to their high stability; however, further verification studies are needed. Moreover, controversial results have been obtained for the same lncRNA in gastric cancer. LncRNA H19 has been demonstrated as a diagnostic biomarker of gastric cancer with a large range of AUC values (0.6–0.98) in recent studies, which may be correlated with the individual differences [83–86]. Further investigations with larger sample size are warranted for improving accuracy and precision. The specific source and molecular mechanisms of lncRNAs in plasma/serum are yet to be determined.

### LncRNAs in gastric juice as diagnostic and prognostic biomarkers of gastric cancer

Gastric juice is directly secreted by the gastric mucosa and can sensitively reflect the pathological state of the stomach, making it an ideal sample for studying gastric cancer [110]. Recent studies have shown that lncRNAs in gastric juice are specific and their expression levels may be inconsistent with those in tissue and plasma. Fei et al. have found that the expression level of LINC00982 is significantly decreased in tumor tissues but increased in gastric juice from patients with gastric cancer [111]. Similar results have been obtained for lncRNA RMRP (RNA component of mitochondrial RNA processing endoribonuclease) and AA174084 by Shao *et al.* [49, 112]. It has been speculated that some lncRNAs may be secreted actively by gastric cancer cells during the disease process or partly by exosomes or other pathways [112].

To date, several lncRNAs, including RMRP, AA174084, PVT1, H19, LINC00982, ABHD11-AS1 (ABHD11 antisense RNA 1), UCA1 (urothelial cancer associated 1), and LINC00152 have been identified from gastric juice and demonstrated as biomarkers for gastric cancer. The sensitivities, specificities, and AUC values of these newly characterized diagnostic biomarkers of gastric cancer range from 41 to 56.4%, 75.4 to 93.4%, and 0.65 to 0.85, respectively. Furthermore, the expression level of AA174084 in gastric juice has been correlated with tumor size, tumor stage, Lauren type, and CEA level in the gastric juice, and a higher AA174084 level in gastric juice indicates a poorer prognosis of gastric cancer patients [112]. The expression level of ABHD11-AS1 in gastric juice has also been associated with the tumor size, tumor stage, and CEA level in the blood, while the high level of ABHD11-AS1 suggests an increased risk of gastric cancer recurrence [113]. Therefore, AA174084 and ABHD11-AS1 can be used for both the diagnosis and prognosis of gastric cancer. In addition, the combination of gastric juice ABHD11-AS1, serum CEA, and gastric juice CEA can improve the diagnostic accuracy of early gastric cancer [113].

In summary, due to the high specificity and reliability, gastric juice lncRNAs can be used as biomarkers for the diagnosis and prognosis of gastric cancer. However, an individual gastric juice lncRNA always has high specificity but insufficient sensitivity as a biomarker. More combination studies, such as the combination of multiple gastric juice lncRNAs, the combination of gastric juice lncRNAs with plasma lncRNAs, and the combination of gastric juice lncRNAs with serum tumor markers may be carried out to increase the sensitivity. Also, further investigations are needed to explore the specific source and molecular mechanisms of gastric juice lncRNAs.

### LncRNA-mediated regulation of chemoresistance in gastric cancer

Chemotherapy is the main treatment option for patients with advanced gastric cancer, while drug resistance is the major cause of gastric cancer treatment failure. The mechanisms of cancer chemoresistance include, but not limited to, drug degradation, amplification and overexpression of oncogenes, anti-apoptosis, immune escape, epithelial-mesenchymal transition (EMT), cancer stemness, autophagy, epigenetic modifications, and up-regulation of multidrug resistance (MDR)-related genes [114–119]. Recent studies have shown that lncRNAs are widely involved in regulating various mechanisms of cancer chemoresistance [120]. LncRNAs have been found to regulate drug resistance by acting as a ceRNA or directly binding to mRNAs or proteins and modulating their expression and/or functions. In this section, we provide a summary of the molecular mechanisms for lncRNAs-mediated gastric cancer chemoresistance (as summarized in Table 2).

**Table 2:** Mechanisms of chemotherapy resistance mediated by lncRNAs

LncRNA	Effection	Drugs	Pathway/target	Mechanism	Refs
MALAT1	Inducing	5-FU, DDP, VCR	miR-23B-3P, ATG12, miR-30b, ATG5, SOX2, nanog	CeRNA, Inducing autophagy, Increasing cancer stemness	[121–123]
AK022798	Inducing	DDP	caspase8, caspase3, MRP1	Inhibiting cell apoptosis, Regulating MDR-related genes	[124]
CRAL	Reversing	DDP	miR-505, CYLD, PI3K/AKT	CeRNA, Promoting DNA damage and apoptosis	[125]
ROR	Inducing	ADR, VCR	MRP1	Inhibiting cell apoptosis, Regulating MDR-related genes	[126]
XLOC_006753	Inducing	5-FU, DDP	PI3K/AKT/mTOR, caspase9, Wnt/β-catenin, Vimentin, Snail	Inhibiting cell apoptosis, Promoting EMT	[127]
MACC1-AS1	Inducing	5-FU, OXA	miR-145-5p, CD133, OCT4, SOX2, LIN28	CeRNA, Increasing cancer stemness	[128]
D63785	Inducing	DOX	miR-422a, MEF2D	CeRNA, Inhibiting cell apoptosis	[129]
LINC01433	Inducing	DOX, DDP	YAP, USP9X	Inhibiting cell apoptosis	[130]
HOXD-AS1	Inducing	DDP	EZH2, PDCD4, H3K27me3	Epigenetically silencing PDCD4 via recruiting EZH2	[131]
HULC	Reversing	DDP, ADM, 5-FU	FOXM1	Suppressing autophagy, Promoting cell apoptosis	[132, 133]
PCAT-1	Inducing	DDP	miR-128, ZEB1, EZH2, PTEN, H3K27me3	CeRNA, Promoting EMT, Epigenetically silencing PTEN via recruiting EZH2	[134, 135]
CASC2	Reversing	DDP	miR-19a	CeRNA, Promoting cell apoptosis	[136]
HOTAIR	Inducing	DDP, 5-FU, ADM, MMC, PTX	miR-17-5p, PTEN, miR-217, miR-34a, PI3K/AKT, Wnt/β-catenin miR-126	CeRNA, Promoting EMT, Regulating MDR-related genes, Inhibiting cell apoptosis, Promoting cell proliferation	[137–140]
THOR	Inducing	DDP	SOX9	Increasing cancer stemness	[141]
BLACAT1	Inducing	OXA	miR-361, ABCB1	CeRNA, Inhibiting apoptosis and promoting invasion, Regulating MDR-related genes	[142]
GHET1	Inducing	DDP	BAK, BCL-2, MDR1, MRP1	Inhibiting cell apoptosis, Regulating MDR-related genes	[143]
ANRIL	Inducing	5-FU, DDP	MDR1, MRP1	Regulating MDR-related genes	[144]
UCA1	Inducing	ADM, DDP, 5-FU	PARP, BCL-2, miR-27b, caspase-3	CeRNA, Inhibiting cell apoptosis	[145, 146]
PVT1	Inducing	PTX, 5-FU, DDP	BCL-2, MDR1, MRP1, mTOR, HIF-1α	Inhibiting cell apoptosis	[147, 148]
MRUL	Inducing	ADM, VCR	ABCB1	Inhibiting cell apoptosis	[149]
SNHG5	Inducing	DDP	BCL-2, BAX, MDR1, MRP1	Inhibiting cell apoptosis, Regulating MDR-related genes	[150]
DANCR	Inducing	DDP	MDR1, MRP1	Inhibiting cell apoptosis, Regulating MDR-related genes	[151]
FAM84B-AS	Inducing	DDP	FAM84B, caspase3, caspase7, caspase9, BCL2, BCL-xL	Inhibiting cell apoptosis	[152]
BCAR4	Inducing	DDP	Wnt/β-catenin, Nanog, OCT3/4, SOX2, c-Myc, KLF4	Increasing cancer stemness	[153]
NEAT1	Inducing	ADM	N/A	Inhibiting cell apoptosis, Promoting invasion	[154]
LEIGC	Reversing	5-FU	CDH1, E-cad, Vimentin, Twist, Slug, ZEB1, Snail	Inhibiting EMT	[155]
CASC9	Inducing	ADM, PTX	MDR1	Inhibiting cell apoptosis, Promoting cell proliferation	[156]
HOTTIP	Inducing	DDP, ADM, 5-FU	E-cad, ZO1, N-cad, Vimentin, ZEB1, Twist	Promoting EMT	[157]
HCP5	Inducing	OXA, 5-FU	MiR-3619-5p, SOX2, OCT4, LIN28, CD1331	CeRNA, Increasing cancer stemness	[158]

*N/A* Not available; *AUC* Area under curve

### LncRNA-mediated cell apoptosis

**Fig. 1:**
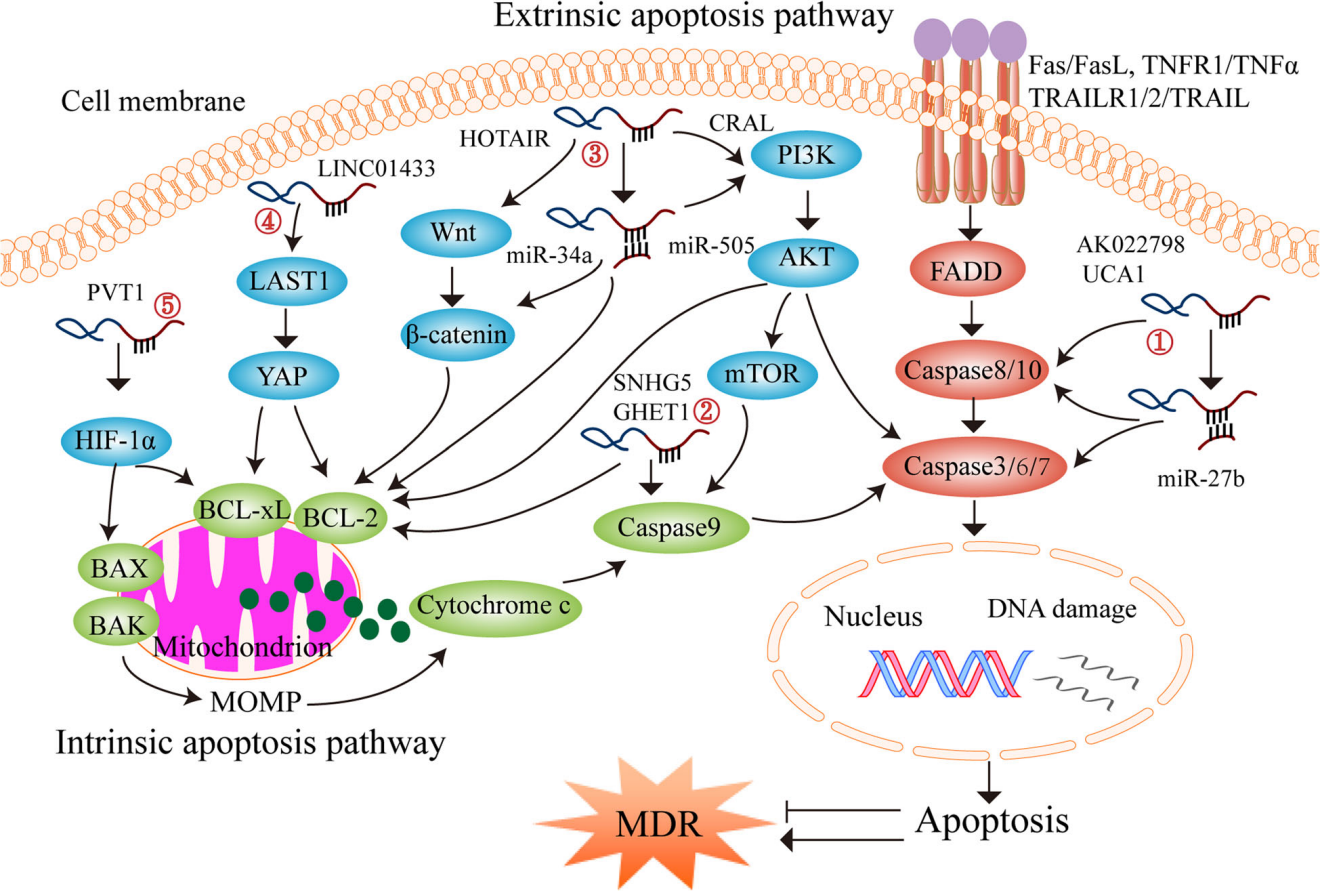
LncRNAs regulate chemoresistance through apoptosis. There are two apoptosis pathways, i.e. extrinsic pathway and intrinsic pathway (mitochondrial pathway). ①-② LncRNAs act as a ceRNA, directly bind to mRNAs or proteins, and regulate multidrug resistance (MDR) through extrinsic and intrinsic pathways of apoptosis. ③-⑤ LncRNAs also regulate apoptosis-mediated MDR through PI3K/AKT, Wnt/β-catenin, Hippo, and HIF-1α signaling pathways

Many anticancer drugs have been found to induce apoptosis and apoptosis-related signaling networks [159, 160]. However, the dysregulation of apoptosis often leads to drug resistance and treatment failure [161]. There are two major pathways of apoptosis, i.e. the extrinsic and intrinsic pathways (mitochondrial pathway) [162, 163]. The extrinsic pathway is initiated by the attachment of death receptors with their death initiating ligands, such as Fas cell surface death receptor (FAS) binding to FAS ligand (FASL), tumor necrosis factor receptor 1 (TNFR1) binding to tumor necrosis factor alpha (TNFα), and TRAIL cell surface receptors 1 and 2 (TRAILR1/2) binding to TNF-related apoptosis-inducing ligand (TRAIL) [163]. Consequently, an adaptor molecule, FAS-associated death domain protein (FADD) couples the death receptors, which leads to the activation of caspase-8 and caspase-10 [163]. Either activated caspase-8 or caspase-10 can directly cleave and activate caspase-3, caspase-6, or caspase-7, thereby promoting apoptosis. Alternatively, irreparable genetic damage, hypoxia, and other internal stimulation can activate apoptosis through the internal mitochondrial pathway. Subsequently, BH3-only protein members, BAX (BCL-2 associated X, apoptosis regulator) and BAK (BCL-2 antagonist/killer), which belong to the B-cell lymphoma-2 (BCL-2) family, can neutralize the anti-apoptotic proteins BCL-2 and BCL-xL (B-cell lymphoma-extra large) [162, 163]. Simultaneously, activation of BAX/BAK can increase the permeability of the mitochondrial outer membrane (MOM) and release different apoptosis mediators, such as cytochrome c, which can activate caspase-9. In turn, caspase-9 cleaves and activates caspase-3, caspase-6, and caspase-7, thus triggering apoptosis [161, 164]. Moreover, PI3K (phosphatidylinositol 3-kinase)/AKT (serine/threonine protein kinase B), Hippo, Wnt/β-catenin, and HIF-1α (hypoxia-inducible factor-1α) signaling pathways are involved in regulating apoptosis. Recent studies have shown that lncRNAs can regulate gastric cancer chemoresistance by modulating these apoptosis-related signaling pathways (Fig. 1).

#### Extrinsic apoptosis pathway

The abnormal expression of caspase-8 and caspase-3 leads to the inhibition of apoptosis and chemotherapy resistance [165]. Hang et al. have reported that the overexpression of lncRNA AK022798 down-regulates the expression of caspase-8 and caspase-3 and inhibits the extrinsic apoptosis pathway, leading to cisplatin (DDP) resistance in gastric cancer cells, while interference with AK022798 increases the expression levels of caspase-8 and caspase-3 and promotes apoptosis, reversing chemotherapy resistance in vitro [124]. Fang et al. have revealed that lncRNA UCA1 functions as a sponge of miR-27b to down-regulate caspase-3 expression and inhibit extrinsic apoptosis pathway, thereby inducing the resistance of gastric cancer cells to DDP, adriamycin (ADR), and 5-fluorouracil (5-FU). It has further been shown that silencing UCA1 increases the expression level of caspase-3, thus promoting apoptosis and reversing MDR in gastric cancer cells in vitro [145, 146].

#### Intrinsic apoptosis pathway (mitochondrial pathway)

The pro-apoptotic proteins (BAX, BAK) and anti-apoptotic proteins (BCL-2, BCL-xL) maintain a dynamic balance in regulating the mitochondrial apoptosis pathway, while the broken balance often causes cancer progression and chemoresistance [166, 167]. Li et al. have shown that lncRNA SNHG5 expression is remarkably higher in DDP-resistant gastric cancer patients and cells [150]. Further mechanism study has revealed that SNHG5 down-regulates BAX expression and up-regulates BCL-2 expression, thereby inhibiting apoptosis and promoting DDP resistance of gastric cancer cells. Similar results have been obtained for lncRNA GHET1 (gastric carcinoma proliferation enhancing transcript 1) by Zhang et al [143]. Moreover, interfering with GHET1 expression causes an increase in BAX level and a decrease in BCL-2 level, thus enhancing the sensitivity of BGC823 and SGC7901 cells to chemotherapy [143]. Du et al. have reported that lncRNA PVT1 inhibits apoptosis and enhances 5-FU resistance of gastric cancer by activating BCL-2 [147]. A Kaplan-Meier analysis has shown that therapy without 5-FU significantly improves the first progression survival and OS of gastric cancer patients with high PVT1 expression, while these patients do not experience survival-related benefits from 5-FU-based chemotherapy [147]. Zhang et al have shown that lncRNA FAM84B-AS (FAM84B antisense RNA) increases the expression levels of BCL-2 and BCL-xL and decreases the expression levels of caspase-9, caspase-3, and caspase-7, consequently inhibiting apoptosis and causing gastric cancer cell resistance to DDP; however, silencing FAM84B-AS enhances gastric cancer cell sensitivity to DDP in vitro and in vivo [152].

#### PI3K/AKT signaling pathway

The PI3K/AKT signaling pathway plays an important role in regulating apoptosis and drug resistance. The activation of PI3K/AKT pathway inhibits apoptosis, leading to tumor progression, drug resistance, and treatment failure, while inhibition of PI3K/AKT signaling reverses drug resistance by inducing apoptosis [168, 169]. In gastric cancer, Wang et al. have reported that CRAL (cisplatin resistance-associated lncRNA) functions as a ceRNA to reverse gastric cancer DDP resistance via the miR-505/CYLD (cylindromatosis)/AKT axis [125]. It has been found that CRAL is mainly located in the cytoplasm and sponges the endogenous miR-505, consequently increasing CYLD expression, suppressing AKT activation, and enhancing the sensitivity of gastric cancer cells to DDP in vitro and in vivo [125]. Zeng et al. have reported that the knockdown of XLOC_006753 can reduce the expression levels of PI3K, p-AKT (Thr308/Ser473), and p-mTOR (phosphorylation mechanistic target of rapamycin kinase), thus activating caspase-9 to promote apoptosis and reverse DDP and 5-FU resistance in gastric cancer cells in vitro [127]. Cheng et al. have demonstrated that lncRNA HOTAIR (HOX transcript antisense RNA) is significantly up-regulated in gastric cancer patients and DDP-resistant cells [137]. HOTAIR has further been found to target miR-34a and activate the PI3K/AKT pathway, consequently decreasing the expression of caspase-3 and BAX, increasing the expression of BCL-2, inhibiting apoptosis, and inducing DDP resistance in gastric cancer cells in vitro and in vivo [137].

#### Hippo signaling pathway

The Hippo signaling pathway is closely associated with apoptosis and MDR by regulating its downstream effectors, Yes-associated protein (YAP) and large tumor suppressor kinase 1 (LATS1) [170]. Recent studies have shown that activation of the Hippo signaling pathway inhibits apoptosis by decreasing the BAX/BCL-2 ratio [171], whereas the downregulation of YAP expression can promote apoptosis [172]. Zhang et al. have shown that LINC01433 decreases the phosphorylation of YAP by disrupting the YAP-LATS1 association. Meanwhile, YAP directly binds to the LINC01433 promoter region and activates its transcription [130]. The formation of the LINC01433-YAP feedback loop suppresses apoptosis and induces resistance to doxorubicin (DOX) and DDP. It has also been found that LINC01433 knockdown significantly increases the sensitivity of gastric cancer cells to DOX and DDP [130].

#### Wnt/β-catenin signaling pathway

Wnt/β-catenin signaling pathway has been demonstrated as an important regulator of cell proliferation, differentiation, and apoptosis, and its abnormal activation is related to MDR in cancer [173, 174]. Targeting the Wnt/β-catenin signaling pathway is a new hope for reversing cancer drug resistance [175, 176]. Cheng et al. have reported that lncRNA HOTAIR directly binds to miR-34a, reduces its expression level, and increases the expression of Wnt and β-catenin [137]. The interference with HOTAIR can decrease the expression of Wnt and β-catenin, thereby increasing the BAX/BCL-2 ratio, activating caspase-3, promoting apoptosis, and reversing DDP resistance in gastric cancer cells in vitro and in vivo [137].

#### HIF-1α signaling pathway

Activation of the HIF-1α signaling pathway is critical for cancer cells adapting to the hypoxic environment, which can mediate apoptosis through the mitochondrial pathway [177]. Recent studies have shown that HIF-1α regulates the mitochondrial apoptosis pathway and MDR by breaking the dynamic balance between the pro-apoptotic proteins (BAX, BAK) and anti-apoptotic proteins (BCL-2, BCL-xL) [178, 179]. Zhang et al. have reported that lncRNA PVT1 is highly expressed in DDP resistant gastric cancer cells and tumor tissues from DDP resistant gastric cancer patients, up-regulates the expression of HIF-1α, inhibits apoptosis, and induces DDP resistance [148]. It has further been shown that silencing PVT1 can reduce the expression of HIF-1α and enhance the sensitivity of gastric cancer cells to DDP [148].

### LncRNA-mediated EMT

**Fig. 2:**
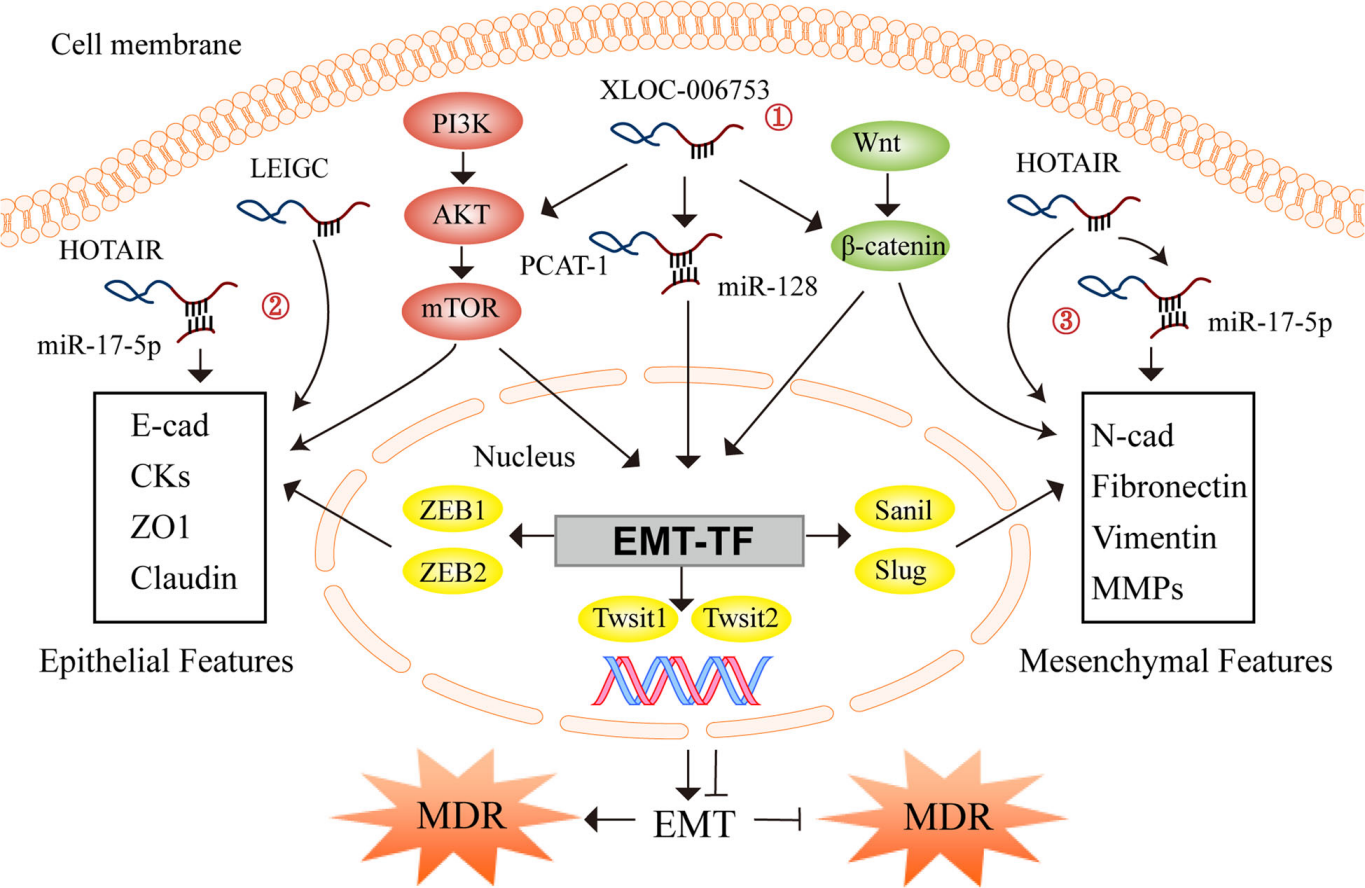
LncRNAs regulate EMT-mediated chemoresistance. ① LncRNAs act as a ceRNA, directly bind to mRNAs or proteins, and regulate EMT-mediated multidrug resistance (MDR) by modulating PI3K/AKT and Wnt/β-catenin signaling pathways. ②-③ LncRNAs also regulate EMT-mediated MDR by targeting EMT markers or transcription factors

EMT is a biological process in which epithelial cells lose their polarity and transform into mesenchymal cells with the ability to move freely [115]. The expression and/or function of epithelial genes such as E-cadherin (E-cad), Claudin, cytokeratins (CKs), and zona occludens 1 (ZO1) are lost during the transition, whereas the expression levels of genes that define the mesenchymal phenotype, such as Vimentin, fibronectin, N-cadherin (N-cad), and matrix metalloproteinases (MMPs) are elevated [180]. The process of EMT is mainly regulated by transcription factors, including zinc-finger-binding transcription factors Snail1 and Snail2, the basic helix-loop-helix (bHLH) factors Twist1 and Twist2, and the zinc-finger E-box-binding homeobox factors ZEB1 and ZEB2 (Fig. 2) [181]. EMT leads to the degradation of adhesion structures between tumor cells, increasing invasiveness and causing chemoresistance and treatment failure [182].

LncRNAs have recently been found to play an important role in the process of drug resistance caused by EMT [183, 184]. LncRNAs regulate EMT-mediated resistance in gastric cancer by regulating EMT markers or transcription factors (Fig. 2). Mao et al. have demonstrated that the expression of lncRNA HOTTIP is up-regulated in MDR gastric cancer cells, which decreases the expression of E-cad and ZO1, increases the expression of N-cad, Vimentin, ZEB1, and Twist, and induces EMT [157]. Conversely, silencing HOTTIP can reverse EMT and enhance the sensitivity of MDR gastric cancer cells to DDP, ADR, and 5-FU in vitro [157]. Han et al. have found that lncRNA LEIGC expression is significantly down-regulated in tumor tissues from human gastric cancer patients, which causes the decreased expression of E-cad and the increased expression of Vimentin, Twist, Slug, ZEB1, and Snail, as well as EMT and resistance of gastric cancer cells to 5-FU [155]. Jia et al. have shown that lncRNA HOTAIR directly targets miR-17-5p to down-regulate E-cad expression and up-regulate the expression of N-cad and Vimentin, thereby inducing EMT and the resistance of gastric cancer cells to DDP, ADR, mitomycin (MMC), and 5-FU [138]. Guo et al. have reported that lncRNA PCAT-1 (prostate cancer associated transcript 1) is highly expressed in DDP-resistant gastric cancer tissues and cells [134]. Mechanistically, PCAT-1 competitively binds to miR-128, upregulates ZEB1 expression, and induces EMT and DDP resistance [134]. Zeng et al. have reported that lncRNA XLOC_006753 expression is up-regulated in gastric cancer tissues and MDR gastric cancer cell lines, and the knockdown of XLOC_006753 can reduce the expression levels of PI3K, p-AKT (Thr308/Ser473), p-mTOR, β-catenin, Vimentin, and Snail, thus reversing EMT and enhancing the sensitivity of gastric cancer cells to DDP and 5-FU in vitro [127].

### LncRNA-mediated cancer cell stemness

**Fig. 3:**
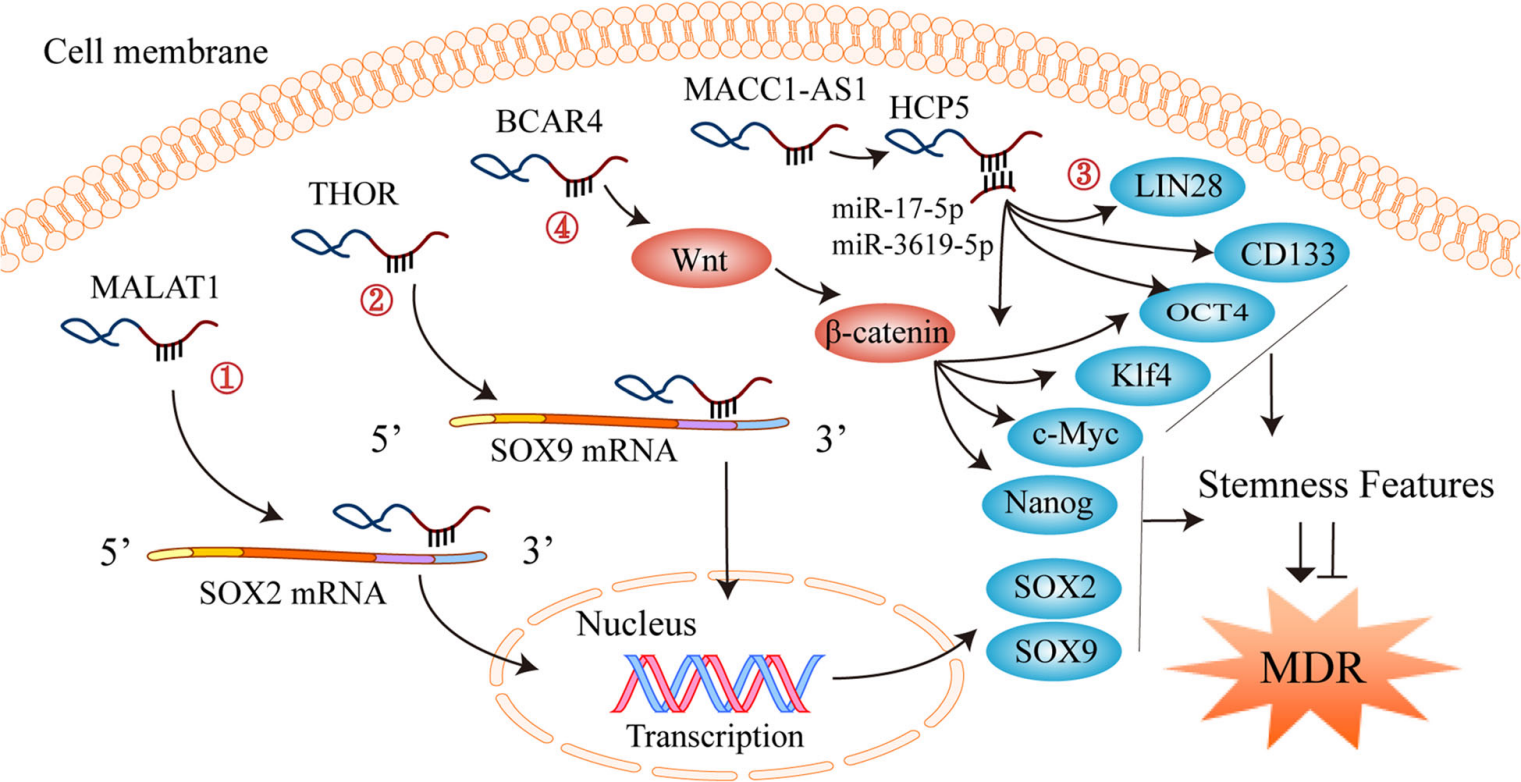
LncRNAs regulate chemoresistance through cancer cell stemness. ①-③ LncRNAs act as a ceRNA, directly bind to mRNAs or proteins, and regulate cancer cell stemness and multidrug resistance (MDR) by modulating stemness-related markers. ④ LncRNAs also regulate cancer cell stemness and MDR through modulating Wnt/β-catenin signaling pathway

Cancer stem cells (CSCs) are a subset of cancer cells with the ability to self-renew and differentiate, which can lead to tumor growth, metastasis, and drug resistance [185]. CSCs play a pivotal role in drug resistance and cancer treatment failure because they have channel proteins to efflux anticancer drugs, which leads to the decreased concentration of drugs in the cells and then induces MDR [185]. The stemness markers of CSCs mainly include cluster of differentiation 24 (CD24), CD29, CD44, CD133, nanog, SRY-box transcription factor 2 (SOX2), SOX9, LIN28, OCT1/2/4, c-Myc, kruppel like factor 4 (KLF4), aldehyde dehydrogenase 1 (ALDH1), and essential specific antigen (ESA) (Fig. 3) [186, 187]. The gain or loss of cancer cell stemness is regulated by the stemness-related pathways and stemness markers [188]. Therefore, targeting the cancer cell stemness-related pathways or markers is an important strategy to reverse drug resistance and enhance drug sensitivity.

LncRNAs have been reported to regulate gastric cancer cell stemness and MDR by modulating stemness-related pathways or markers (Fig. 3). Activation of the Wnt/β-catenin pathway has been found to promote the stemness of cancer cells [189, 190]. Wang et al. have reported that lncRNA BCAR4 (breast cancer anti-estrogen resistance 4) is highly expressed in DDP-resistant gastric cancer cells. Further studies have shown that BCAR4 activates the Wnt/β-catenin signaling pathway and up-regulates the expression of stemness markers nanog, OCT3/4, SOX2, c-Myc, and KLF4, which further enhance gastric cancer cell stemness and DDP resistance [153]. He et al. have found that lncRNA MACC1-AS1 (MACC1 antisense RNA 1) competitively antagonizes miR-145-5p, thereby up-regulating the levels of diacylglycerol cholinephosphotransferase (CPT1) and acetyl-CoA synthetase (ACS) to participate in fatty acid oxidation (FAO), increasing the expression of CD133, OCT4, SOX2, and LIN28, and inducing the resistance of gastric cancer cells to 5-FU and oxaliplatin (OXA) [128]. Unsurprisingly, the knockdown of MACC1-AS1 attenuates the stemness of gastric cancer cells and reverses MDR [128]. Song et al. have shown that THOR (testis-associated highly conserved oncogenic long non-coding RNA) is highly expressed in gastric cancer tissues and cells, whereas THOR knockdown decreases the expression of SOX9 through directly binding to its 3′UTR, thus inhibiting gastric cancer cell stemness and reversing the resistance of gastric cancer cells to DDP [141]. Xiao et al. have demonstrated that lncRNA MALAT1 directly binds to *SOX2* mRNA, enhances its stability, and increases its expression, which further promotes the stemness of gastric cancer cells and induces DDP resistance [121]. Wu et al. have found that lncRNA HCP5 (histocompatibility leukocyte antigen complex P5) drives FAO by sponging miR-3619-5p and promoting stemness and the resistance of gastric cancer cells to 5-FU and OXA [158].

### LncRNA-mediated autophagy

**Fig. 4:**
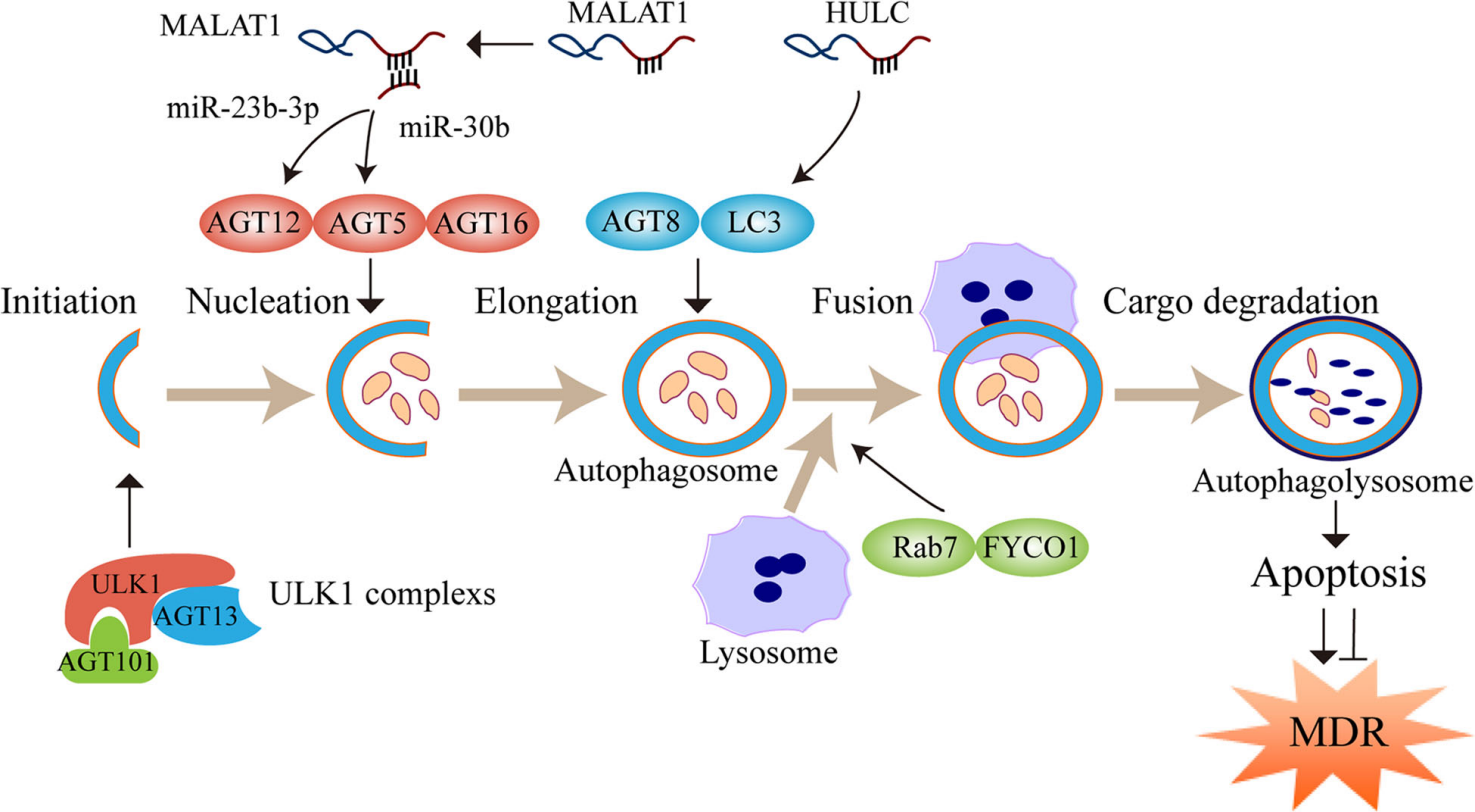
LncRNAs regulate autophagy-mediated chemoresistance. The process of autophagy is divided into five distinct stages: initiation, vesicle nucleation, vesicle elongation, vesicle fusion, and cargo degradation. LncRNAs act as a ceRNA, directly bind to mRNAs or proteins, and regulate autophagy-mediated multidrug resistance (MDR) by targeting ATGs or ATG-LC3 complex

Autophagy is an evolutionarily conserved cellular process, through which damaged organelles and superfluous proteins are degraded, thereby maintaining the correct cellular balance [191]. The process of autophagy is divided into five distinct stages (Fig. 4): 1) initiation, 2) vesicle nucleation, 3) vesicle elongation, 4) vesicle fusion, and 5) cargo degradation [192]. Firstly, various stresses (deficiency of oxygen, ultraviolet rays, or exposure to toxic agents) trigger autophagy, and then the assembly of the Unc-51-like kinase 1 (ULK1) complex, comprising ULK1, autophagy-related genes 13 (ATG13), and ATG101 induces nucleation of the autophagy-isolation membrane. Following nucleation, the elongation of the isolation membrane is regulated by the ATG12-ATG5-ATG16 complex. Moreover, the isolation membrane collects cellular materials to degrade and form an autophagosome, which is regulated by ATG8/LC3 (microtubule-associated protein 1A/1B-light chain 3) complex. Subsequently, the autophagolysosome is formed through the fusion of autophagosome and lysosome, which is mediated by Ras-related protein 7 (Rab7) and FYVE and coiled-coil protein 1 (FYCO1) transport proteins. Finally, cellular components are degraded and recycled to supply energy to the cells due to the action of hydrolytic enzymes [193].

Recent studies have shown a paradoxical role of autophagy in cancer [194]. Autophagy is a double-edged sword of cancer MDR; it not only participates in the development of MDR and protects cancer cells from chemotherapy but also promotes cell death and mediates chemosensitization in MDR cancer cells with insufficient apoptosis [195, 196]. In gastric cancer, lncRNAs are widely involved in regulating various stages of autophagy as well as autophagy-mediated MDR (Fig. 4). Hu et al. have demonstrated that lncRNA MALAT1 acts as a ceRNA for miR-23b-3p and attenuates the inhibitory effects of miR-23b-3p on ATG12 expression, thus inducing autophagy-mediated resistance of gastric cancer cells to DDP and vincristine (VCR) in vitro and in vivo [122]. It has been found that MALAT1 is highly expressed in DDP-resistant AGS and HGC-27 cells [122, 123]. MALAT1 also binds to miR-30b and increases ATG5 expression, whereas MALAT1 knockdown can suppress autophagy and enhance the chemosensitivity of gastric cancer cells [122, 123]. Xin et al have found that lncRNA HULC interacts with forkhead box M1 (FOXM1) and stabilizes this protein, thus increasing the ratio of LC3-II/LC3-I and inducing autophagy-mediated DDP resistance [132]. As expected, silencing HULC has been shown to inhibit autophagy and enhance chemotherapy sensitivity of gastric cancer cells in vitro and in vivo [132].

### LncRNA regulates MDR-related genes

**Fig. 5:**
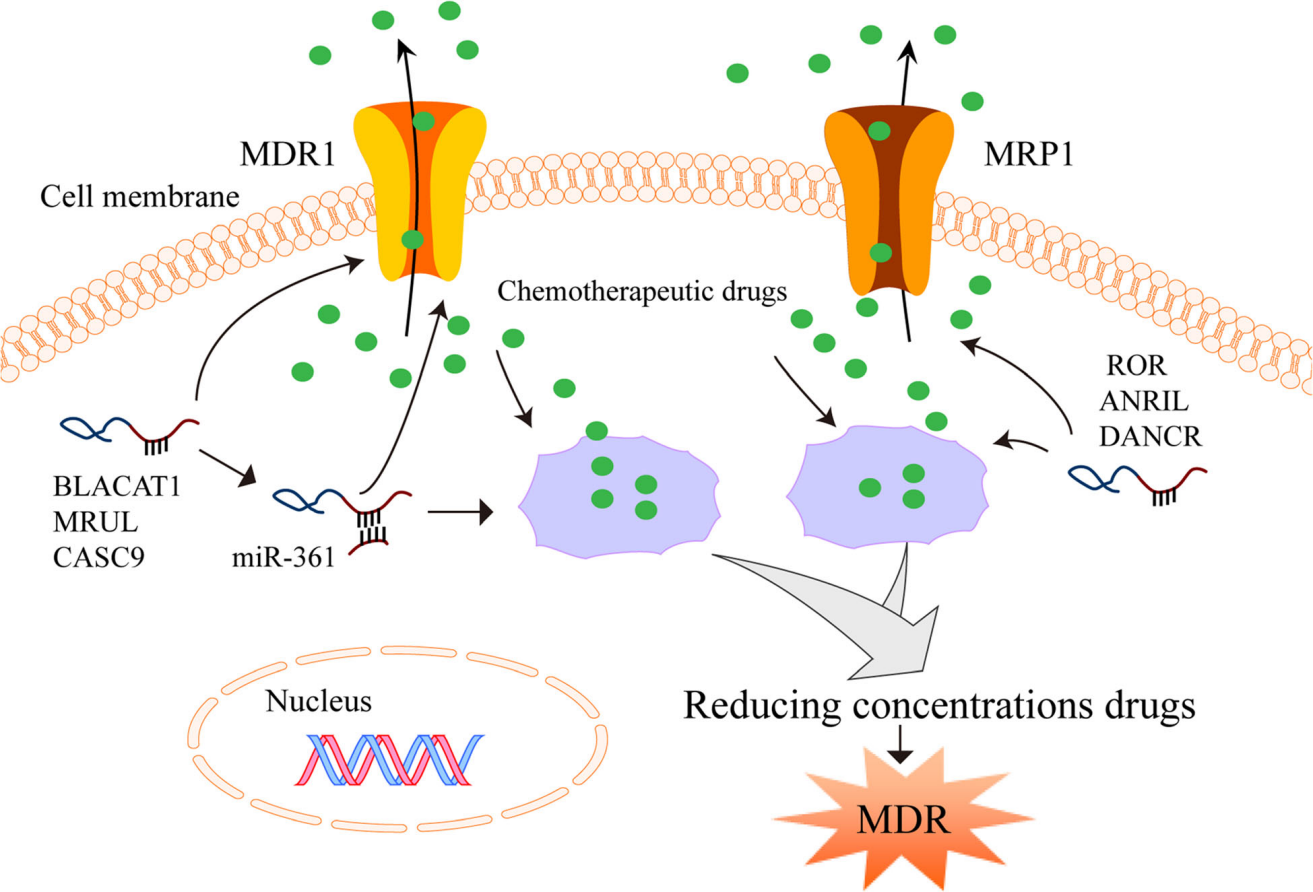
LncRNAs regulate chemoresistance through modulating MDR-related genes**.** The ABC transporters export chemotherapy drugs out of the cells, leading to resistance with reduced concentrations of the drugs intracellularly. The transporters also sequestrate intracellular drugs into membrane vesicles in the cytoplasm, resulting in chemotherapy resistance. LncRNAs can act as a ceRNA, directly bind to mRNAs or proteins, and regulate MDR through up-regulating the expression of MDR-related genes (MDR1 and MRP1)

Ample evidence suggests that the expression of ATP-binding cassette (ABC) transporters, especially multidrug resistance protein 1 (MDR1, also known as P-glycoprotein or P-gp) and multidrug resistance-associated protein 1 (MRP1), which are encoded by the ABC subfamily B member 1 (ABCB1) and the ABC subfamily C member 1 (ABCC1), respectively, confers resistance to chemotherapy [197]. The ABC transporters export chemotherapeutic drugs out of the cells, resulting in resistance with reduced concentrations of the drugs intracellularly. The transporters sequestrate intracellular drugs into membrane vesicles in the cytoplasm, which also causes chemotherapy resistance (Fig. 5) [198].

In gastric cancer, lncRNAs participate in the acquisition of chemotherapy resistance by regulating MDR-related genes (Fig. 5). Wang et al have shown that lncRNA ROR (regulator of reprogramming) expression is positively associated with MDR and poor prognosis of patients with gastric cancer [126]. It has also been reported that ROR depletion reduces MRP1 expression and reverses resistance to ADR and VCR [126]. Wu et al have reported that lncRNA BLACAT1 (bladder cancer associated transcript 1) accelerates the OXA-resistance acquisition of gastric cancer cells by targeting miR-361 and increasing MDR1 protein expression in vitro and in vivo [142]. Moreover, Wang et al have demonstrated that MRUL (MDR-related and upregulated lncRNA) exerts an enhancer-like role in up-regulating MDR1 expression, whereas MRUL knockdown reduces MDR1 expression and reverses resistance to ADR and VCR in vitro and in vivo [149]. Shang et al have found that lncRNA CASC9 (cancer susceptibility 9) is overexpressed in BGC823 and SGC7901 cells that are resistant to paclitaxel (PTX) or ADR [156]. Further studies have shown that CASC9 knockdown decreases MDR1 expression and restores the sensitivity of gastric cancer cells to PTX and ADR in vitro [156]. It has also been found that lncRNA ANRIL (antisense noncoding RNA in the INK4 locus) is highly expressed in DDP-resistant and 5-FU-resistant gastric cancer tissues and cells [144]. Importantly, ANRIL expression is positively correlated with the expression of MDR1 and MRP1 while ANRIL knockdown down-regulates the expression of MDR1 and MRP1 and reverses MDR [144]. Xu et al have shown that the overexpression of lncRNA DANCR up-regulates the expression of MDR1 and MRP1 and induces DDP resistance of gastric cancer cells in vitro [151]*.*

### LncRNA-mediated epigenetic modifications

Epigenetic modifications of histones can regulate resistance to anticancer drugs because cancer cells can develop drug resistance by reprogramming epigenetic networks to maintain their intrinsic homeostasis [199]. For example, the demethylation of H3K4 promotes DDP resistance of cancer cells while restoration of H3K4 methylation reverses such resistance [200]. Further, histone deacetylases regulate the functional equilibrium of histone acetylation and deacetylation, and its dysfunction leads to chemotherapy resistance [201].

In gastric cancer, lncRNAs also contribute to chemotherapy resistance by regulating histone methylation. Ye et al have found that lncRNA HOXD-AS1 (HOXD antisense RNA 1) is highly expressed in DDP-resistant gastric cancer tissues and cells [131]. Mechanism studies have shown that HOXD-AS1 epigenetically inhibits PDCD4 expression by binding to the histone methyltransferase enhancer of zeste homologue 2 (EZH2) on the promoter of *PDCD4*, thus increasing H3K27me3 level and inducing DDP resistance in gastric cancer cells [131]. Li et al have shown that lncRNA PCAT-1 epigenetically silences phosphatase and tensin homolog (PTEN) by binding to EZH2, which also increases H3K27me3 level and causes DDP resistance [135]. More importantly, the knockdown of either HOXD-AS1 or PCAT-1 enhances the sensitivity of DDP-resistant gastric cancer cells to DDP.

## Perspectives and future directions

Overall, this review provides compelling evidence for lncRNAs as biomarkers for diagnosis, prognosis, and regulator of chemoresistance in gastric cancer. Because lncRNAs in the circulation (serum/plasma) or gastric juice are easy to obtain with non-invasive methods, they have great advantages as biomarkers for early screening, diagnosis, and prognosis of gastric cancer. Currently, lncRNA PCA3 in urine has been used as an early screening biomarker of prostate cancer [56, 57]. Therefore, it is of great clinical value to validate lncRNAs in serum/plasma or gastric juice as biomarkers for gastric cancer. Considering that most of the studies cited in this review are single-center trials with small samples, the results may be biased. Next, more in-depth studies are needed to accelerate the clinical applications of lncRNAs, such as increasing the sample size or conducting multi-center research to reduce the errors caused by individual differences.

LncRNAs are also involved in the regulation of chemotherapy resistance by modulating the signaling pathways related to apoptosis, EMT, cancer cell stemness, and autophagy, the expression of MDR-related genes, and epigenetic modifications. Therefore, targeting lncRNAs may be a promising strategy to enhance chemosensitivity and improve the efficacy of gastric cancer chemotherapy [202]. Previous studies have shown that treatment without 5-FU significantly improves the first progression survival and overall survival of gastric cancer patients with high PVT1 expression [147]. However, the patients harboring PVT1 overexpression do not obtain survival-related benefits from 5-FU-based chemotherapy [147]. Therefore, it is of great importance to further characterize lncRNAs in liquid biopsies as a guide to precision medicine for gastric cancer patients.

There is an increasing interest in targeting lncRNAs for gastric cancer therapy. However, concerns have also been raised about the therapeutic potential of targeting a single lncRNA and the current targeting strategies. First of all, despite the great progress in understanding the structures and functions of lncRNAs since their discovery, the study of lncRNAs is still a burgeoning research field and we have only touched on the tip of this iceberg. Furthermore, given the large number of lncRNAs and their up-regulation or down-regulation in gastric cancer, it is critically needed to determine the most clinically relevant lncRNAs in this disease. Of note, lncRNAs are poorly conserved among different species. Therefore, the lncRNA-targeting strategies that are developed by utilizing various animal models and cell culture systems cannot be easily extended to human applications. The latest advances in CRISPR (clustered regularly interspaced short palindromic repeats)/Cas9 gene knockout, knock-in, and point mutations may help to understand the biological role of lncRNAs. At the same time, the development of human primary cell models and patient-derived tumor xenograft (PDX) animal models may be helpful for investigating the role of lncRNAs and developing lncRNA-targeting strategies. In the near future, the development of lncRNA-targeted cancer therapy seems to be very promising.

In conclusion, accumulating evidence has shown the potential of lncRNAs as biomarkers in liquid biopsies throughout the entire management process of gastric cancer, including diagnosis, selection of chemotherapeutics, monitoring of curative effects, and prognosis.

## Data Availability

Not applicable.

## Abbreviations

5-FU: 5-fluorouracil
ABC: ATP-binding cassette
ABCB1: ABC subfamily B member 1
ABCC1: ABC subfamily C member 1
ABHD11: Abhydrolase domain containing 11
ABHD11-AS1: ABHD11 antisense RNA 1
ACS: Acetyl-CoA synthetase
ADR: Adriamycin
AFP: Alpha fetoprotein
AKT: serine/threonine protein kinase B
ALDH1: Aldehyde dehydrogenase 1
ANRIL: Antisense noncoding RNA in the INK4 locus
AOC4P: Amine oxidase copper containing 4, pseudogene
ARHGAP27P1: Rho GTPase activating protein 27 pseudogene 1
ATG: Autophagy-related genes
AUC: Area under curve
B3GALT5: Beta-1,3-galactosyltransferase 5
B3GALT5-AS1: B3GALT5 antisense RNA 1
BAK: BCL-2 antagonist/killer
BANCR: BRAF-activated non-protein coding RNA
BAX: BCL-2 associated X, apoptosis regulator
BCAR4: Breast cancer anti-estrogen resistance 4
BCL-2: B-cell lymphoma-2
BCL-xL: B-cell lymphoma-extra large
BLACAT1: Bladder cancer associated transcript 1
C5orf66-AS1: C5orf66 antisense RNA 1
CA: Carbohydrate antigen
CASC: Cancer susceptibility
CCAT2: Colon cancer associated transcript 2
CD: Cluster of differentiation
CDH1: Cadherin 1
CEA: Carcinoembryonic antigen
CEBPA: CCAAT enhancer binding protein alpha
CEBPA-AS1: CEBPA divergent transcript
CeRNA: Competitive endogenous RNA
circRNAs: Circular RNAs
CKs: Cytokeratins
c-Myc/MYC: MYC proto-oncogene, bHLH transcription factor
CPT1: Diacylglycerol cholinephosphotransferase
CRAL: Cisplatin resistance-associated lncRNA
CRISPR: Clustered regularly interspaced short palindromic repeats
CSCs: Cancer stem cells
CTDHUT: CTD highly upregulated transcript
CYLD: Cylindromatosis
DANCR: Differentiation antagonizing non-protein coding RNA
DDP: Cisplatin
DGCR5: DiGeorge syndrome critical region gene 5
DOX: Doxorubicin
E-cad: E-cadherin
EMT: Epithelial-mesenchymal transition
ESA: Essential specific antigen
EZH2: Enhancer of zeste homologue 2
FADD: FAS-associated death domain protein
FAM49B: Family with sequence similarity 49, member B
FAM49B-AS: FAM49B antisense RNA
FAM84B: Family with sequence similarity 84, member B
FAM84B-AS: FAM84B antisense RNA
FAO: Fatty acid oxidation
FAS: Fas cell surface death receptor
FASL: FAS ligand
FOXD2: Forkhead box D2
FOXD2-AS1: FOXD2 adjacent opposite strand RNA 1
FOXFOXMM1: Forkhead box M1
FYCO1: FYVE and coiled-coil protein 1
GACAT2: Gastric cancer associated transcript 2
GASL1: Growth arrest associated lncRNA 1
GHET1: Gastric carcinoma proliferation enhancing transcript 1
GNAQ-6:1: G protein subunit alpha q-6:1
GUSBP11: GUSB pseudogene 11
H19: H19 imprinted maternally expressed transcript
HCP5: Histocompatibility leukocyte antigen complex P5
HIF-1α: Hypoxia-inducible factor-1α
HOTAIR: HOX transcript antisense RNA
HOTTIP: HOXA distal transcript antisense RNA
HOXA11: Homeobox A11
HOXA11-AS: HOXA11 antisense RNA
HOXD: Homeobox D cluster
HOXD-AS1: HOXD antisense RNA
HULC: Hepatocellular carcinoma upregulated long noncoding RNA
INHBA: Inhibin subunit beta A
INHBA-AS1: INHBA antisense RNA 1
KLF4: Kruppel Like Factor 4
LATS1: Large tumor suppressor kinase 1
LC3: Microtubule associated protein 1 light chain 3 alpha
LEIGC: LncRNA chr2:118381039–118,383,698
LincRNAs: Intergenic lncRNAs
LncRNAs: Long non-coding RNAs
MACC1: MET transcriptional regulator MACC1
MACC1-AS1: MACC1 antisense RNA 1
MALAT1: Metastasis associated lung adenocarcinoma transcript 1
MDR: Multidrug resistance
MDR1: Multidrug resistance protein 1
MEF2C: Myocyte enhancer factor 2C
MEF2C-AS1: MEF2C antisense RNA 1
MEF2D: Myocyte enhancer factor 2D
MEG3: Maternally expressed 3
MIR4435–2HG: MIR4435–2 host gene
MIAT: Myocardial infarction associated transcript
miRNAs: microRNAs
MMC: Mitomycin
MMPs: Matrix metalloproteinases
MNX1: Motor neuron and pancreas homeobox 1
MNX1-AS1: MNX1 antisense RNA 1
MOM: Mitochondrial outer membrane
MOMP: Mitochondrial outer membrane permeability
MRP1: Multidrug resistance-associated protein 1
MRUL: MDR-related and upregulated lncRNA
MT1JP: Metallothionein 1 J, pseudogene
mTOR: Mechanistic target of rapamycin kinase
N-cad: N-cadherin
ncRNAs: Noncoding RNAs
NEAT1: Nuclear paraspeckle assembly transcript 1
NSE: Neuron-specific enolase
OCT: Organic cation/carnitine transporter
OS: Overall survival
OXA: Oxaliplatin
PANDAR: Promoter of CDKN1A antisense DNA damage activated RNA
PARP: Poly-(ADP-ribose) polymerase
PCA3: Prostate cancer associated 3
PCAT1: Prostate cancer associated transcript 1
PDCD4: Programmed cell death 4
PDX: Patient-derived tumor xenograft
P-gp: P-glycoprotein
PI3K: Phosphatidylinositol 3-kinase
PCSK2–2:1: Proprotein convertase subtilisin/kexin type 2–2:1
PTCSC3: Papillary thyroid carcinoma susceptibility candidate 3
PTEN: Phosphatase and tensin homolog
PTX: Paclitaxel
PVT1: Plasmacytoma variant translocation 1
Rab7: Ras-related protein 7
RMRP: RNA component of mitochondrial RNA processing endoribonuclease
ROC: Receiver operator characteristic curve
ROR: Regulator of reprogramming
SMARCC2: SWI/SNF related, matrix associated, actin dependent regulator of chromatin subfamily c member 2
SNHG: Small nucleolar RNA host gene
snoRNAs: Small nucleolar RNAs
SOX: SRY-box transcription factor
THOR: Testis-associated highly conserved oncogenic long non-coding RNA
TINCR: Terminal differentiation-induced ncRNA
TNFR1: Tumor necrosis factor receptor 1
TNFα: Tumor necrosis factor alpha
TNM: Tumor, node, metastasis
TRAIL: TNF-related apoptosis-inducing ligand
TRAILR1/2: TRAIL cell surface receptors 1 and 2
TUBA4B: Tubulin alpha 4b
TUG1: Taurine up-regulated 1
UCA1: Urothelial cancer associated 1
UEGC1: ENST00000568893.1
UEGC2: ENST00000378432.1
ULK1: Unc-51-like kinase 1
USP9X: Ubiquitin specific peptidase 9 X-linked
UTR: Untranslated regions
VCR: Vincristine
XIST: X inactive specific transcript
YAP: Yes-associated protein
ZEB1: Zinc finger E-box binding homeobox 1
ZFAS1: ZNFX1 antisense RNA 1
ZNFX1: Zinc finger NFX1-type containing 1
ZO1: Zona occludens 1
